# In Vitro Characterization of a Nineteenth-Century Therapy for Smallpox

**DOI:** 10.1371/journal.pone.0032610

**Published:** 2012-03-09

**Authors:** William Arndt, Chandra Mitnik, Karen L. Denzler, Stacy White, Robert Waters, Bertram L. Jacobs, Yvan Rochon, Victoria A. Olson, Inger K. Damon, Jeffrey O. Langland

**Affiliations:** 1 Biodesign Institute, Arizona State University, Tempe, Arizona, United States of America; 2 Department of Naturopathic Research, Southwest College of Naturopathic Medicine, Tempe, Arizona, United States of America; 3 Herbal Vitality, Inc., Sedona, Arizona, United States of America; 4 Division of Viral and Rickettsial Diseases, National Center for Emerging and Zoonotic Infectious Diseases, Centers for Disease Control and Prevention, Atlanta, Georgia, United States of America; Vanderbilt University, United States of America

## Abstract

In the nineteenth century, smallpox ravaged through the United States and Canada. At this time, a botanical preparation, derived from the carnivorous plant *Sarracenia purpurea*, was proclaimed as being a successful therapy for smallpox infections. The work described characterizes the antipoxvirus activity associated with this botanical extract against vaccinia virus, monkeypox virus and variola virus, the causative agent of smallpox. Our work demonstrates the *in vitro* characterization of *Sarracenia purpurea* as the first effective inhibitor of poxvirus replication at the level of early viral transcription. With the renewed threat of poxvirus-related infections, our results indicate *Sarracenia purpurea* may act as another defensive measure against *Orthopoxvirus* infections.

## Introduction

In the late 1800's, the Micmac Indians of Nova Scotia proclaimed the existence of a botanical-based remedy for smallpox. During this time, Herbert Miles, the Assistant Surgeon to the Royal Artillery, reported that during an outbreak of smallpox “an old squaw going amongst them, and treating the cases with (a botanical) infusion…was so successful as to cure every case”. This botanical infusion was later described as being derived from the carnivorous plant, *Sarracenia purpurea* [1], [2].

In 1892, Charles Millspaugh described that the Native Americans of eastern North America used *S. purpurea* as a poultice against smallpox and it provided “the greatest remedy known for the dreadful scourge” [3]. C.G. Logie, Surgeon-Major of the Royal Horse Guards, treated variola-infected men in his regiment and found that *S. purpurea* “seemed to arrest the development of the pustules, killing, as it were, the virus from within, thereby changing the character of the disease and doing away with the cause of pitting” [4]. However, the putative medicinal properties of *S. purpurea* have been largely forgotten.

Before human development, *S. purpurea*, also known as Huntsman's Cup, Side-saddle flower or Indian Pitcher Plant, was widely distributed ranging from Labrador to Florida along the Atlantic seacoast of North America and westward to Wisconsin and Minnesota [13], [14]. *S. purpurea* is an insectivorous bog plant, the leaves of which form cups and become filled with water to capture insects.

Smallpox outbreaks have occurred within the human population for thousands of years, but the natural disease is now considered eradicated after a successful worldwide vaccination program. In recent years there has been a heightened concern that variola virus might be used as an agent of bioterrorism [30]. In addition, the related monkeypox virus represents an emerging threat to the human population [19], [30]. Though the vaccine provides effective protection against poxvirus infection, side effects and risks associated with the smallpox vaccine are reasonably common and can be quite severe [27], [30]. The increasing threat of *Orthopoxvirus-*related infections highlights the need to discover effective poxvirus countermeasures. In this manuscript we present the ‘rediscovery’ and characterization of *S. purpurea* as an effective inhibitor of *Orthopoxvirus* replication.

## Methods

### Cell lines and viruses

Rabbit Kidney (RK-13, American Type Culture Collection (ATCC) CCL-37) cells were maintained in Eagle's Minimal Essential Medium (MEM) (Cellgro) supplemented with 5% fetal bovine serum (FBS) (HyClone). HeLa (ATCC CCL-2) were maintained in Dulbecco's Modified-Minimal Essential Medium (DMEM) (Cellgro) supplemented with 5% FBS. All cells were incubated at 37°C in the presence of 10% CO_2_. Vaccinia virus (Copenhagen strain), designated as VACV, was provided by Virogenetics. Monkeypox virus (Walter Reed 267 strain), designated as MPXV, was provided by BEI Resources. Variola virus (BSH74 sol strain), designated as VARV, was provided and used for experiments at the Centers for Disease Control and Prevention (Atlanta, GA). Adenovirus Type 5 (Adeno), vesicular stomatitis virus, Indiana strain (VSV), mouse hepatitis virus A59 (MHV), reovirus Type 3, Dearing strain (Reo), encephalomyocarditis virus (EMCV) were obtained from American Type Culture Collection (ATCC). For MHV, HeLa-MHVR cells expressing the MHV A59 CEACAM (carcinoembryonic antigen cell adhesion molecule isoform 1a) receptor were kindly provided by Tom Gallagher (Loyala University Medical Center, Maywood, IL).

### Botanical extract preparation

The following herbs were used in this study: *Sarracenia purpurea*, *Astragalus membranaceus*, *Echinacea angustifolia*, and *Coriolus versicolor.* For the preparation of *S. purpurea* extract, fresh whole plants grown in a greenhouse in the Southeastern United States were shipped overnight express and received at the manufacturing facility (Sedona, AZ). The plants were manually cleaned on the same day, with special attention to cleaning the base portion of the plant's pitcher structure so that it was free from contamination with forest detritus. Cleaned whole plant was ground gently in a Hamilton Beach Stainless steel blender in the presence of a blend of 190-proof grain ethanol/distilled water/vegetable glycerin (63%/32%/5%). The plant/liquid mixture was transferred to an amber colored glass container, sealed tightly, and incubated at room temperature for 48 days. The liquid was pressed from the solid plant material, filtered through unbleached paper filters, pooled, and bottled in amber colored glass bottles. Fresh *Echinacea angustifolia* roots were harvested from central Kansas. All fresh plants were shipped overnight express to the manufacturing facility for immediate validation and processing. Fresh plant material was validated using taxonomic keys. Dried *Astragalus membranaceus* root slices were purchased from Mayway Corporation (Oakland, CA). Dried plant material was validated using herbal pharmacopoeia monographs. Fresh plants were mixed with distilled water/190 proof ethanol/glycerol at a ratio 1∶3 (*Echinacea*) [weight of botanical to volume of liquid], ground gently in a 1 gallon stainless steel Hamilton Beach blender, and the herb-liquid mixture was transferred to a clean amber colored gallon glass jar and sealed. Dried botanicals were ground in a 1 gallon stainless steel Hamilton Beach blender, transferred to a clean amber colored gallon glass jar, and a mixture of distilled water/190 proof ethanol/glycerol was added at a ratio of 1∶5 (weight of botanical to volume of liquid). Distilled water/190 proof ethanol/glycerol concentrations were as followed (*Astragalus* 74/26/0; *Echinacea* 48/47/5). The mixtures were kept at room temperature for 2 to 6 weeks, followed by separation of the liquid portion from the solid herb portion using a mechanical press. The extracted liquid was filtered using unbleached paper filters, pooled, and dispensed in amber colored bottles. A sample of each extract was dried and all extracts were found to contain similar concentrations of non-volatile solutes (ranging between 62.4–136.8 mg/ml extract).

### Cidofovir treatment

Cidofovir (Sigma-Aldrich) treatment was done as previously described [15], [16].

### VACV plaque assay

RK-13 cells were infected with 150 pfu of VACV. At 15 mpi, the virus was removed and 0, 1, 3, 10, and 30 microL of *S. purpurea* extract per mL of cell culture media was added. For the cells receiving multiple *S. purpurea* treatments, media was replaced with fresh media containing the varying amounts of *S. purpurea* extract every six hours. Cells were incubated at 37°C in the presence of 5% CO_2_ for 48 hours. VACV plaques were visualized by crystal violet staining [23].

### Single-cycle growth kinetics

RK-13 cells were infected with VACV at an MOI of 10 for 15 min, washed 2 times with media, and treated with 25 microL *S. purpurea* extract/mL media. For the cells receiving multiple *S. purpurea* treatments, media was replaced with fresh media containing the varying amounts of *S. purpurea* extract every six hours. Cells not treated with *S. purpurea*, were treated with 25 microL 63% ethanol, 5% glycerol solution (ethanol/glycerol carrier)/mL media. Infected cells were harvested at 2, 6, 12, 18 and 24 hpi by scraping into the media and freeze/thawing 3 times. Viral titers were determined by plaque assay and crystal violet staining in RK-13 cells.

### *Orthopoxvirus* protein synthesis and CPE

HeLa cells were infected with VACV at an MOI = 10 for 15 minutes, washed 2× with media and treated with 25 microL *S. purpurea* extract/mL media at 0, 15, 30, 60, and 120 mpi. Cell monolayers were photographed at 6 hpi to record VACV-induced CPE. At 3 and 6 hpi cell lysates were prepared by RIPA lysis [24]. Cell lysates were analyzed on 12% polyacrylamide gels by SDS-PAGE. Proteins were transferred nitrocellulose membranes, incubated with antibodies directed against the VACV-E3L protein or against total VACV proteins, and detected by chemiluminescence (Pierce SuperSignal West Pico Chemiluminescence Substrate). The relative levels of E3L protein were quantified using ImageQuant software. MPXV infections were done under the same conditions using a BSL-3 facility and treating with *S. purpurea* at 0 and 15 mpi. VARV infections were done at the Centers for Disease Control and Prevention (Atlanta, GA) under similar conditions using a BSL-4 facility and treating with *S. purpurea* at 0 mpi. Infections with Adeno, VSV, MHV, and Reo viruses were done similarly with *S. purpurea* treatment at 15 mpi and lysates prepared at 8 hpi. Antibodies specific for the Adeno-E1, VSV-G, MHV-E and Reo-core proteins were used to measure viral protein synthesis. Treatment of VACV with other botanical extracts (*Echinacea*, *Astragalus*, *Coriolus*, and *Glycyrrhiza*) were done at 15 mpi at 25 microL extract/mL media and cell lysates prepared at 6 hpi.

### Cell viability

HeLa cells were treated with *S. purpurea* at the concentrations indicated. Viability was determined by trypan blue exclusion at 6 hours post treatment.

### Immunofluorescence

Subconfluent HeLa cells, grown on poly-l-lysine coated coverslips, were infected with a VACV construct in which the cyan fluorescent protein was fused to the core A5 protein (kindly provided by B. Moss, NIH) at an MOI = 20. The infection was kept at 4°C or room temperature for 10 minutes, washed 2-times with media, and treated with *S. purpurea* extract. After 1 hour of treatment, cells were rinsed with PBS and fixed with 4% paraformaldehyde for 20 minutes at room temperature. The cells were quenched with 50 mM ammonium acetate in PBS for 10 minutes at room temperature. The cells were rinsed with PBS and permeabilized with 0.2% Triton X-100 for 15 minutes at room temperature. The cells were blocked with blocking buffer GTP (0.2% gelatin/0.1% Triton X-100/1XPBS) for 30 minutes followed by overnight staining with eIF2-alpha antiserum (Cell Signaling) at 1∶500 dilution at 4°C. Secondary antibodies (Alexa Fluor 488- Invitrogen) were applied to the coverslips at 1∶500 in GTP for 1 hour at room temperature followed by 3 washes of GTP. The coverslips were mounted in ProLong Gold antifade reagent (Invitrogen) and samples analyzed using Zeiss Duo confocal microscope.

### VACV *in vivo* protein labeling

HeLa cells were infected by VACV at an MOI of 10 for 15 minutes, washed 2× with media, and treated with 25 microL *S. purpurea* extract/mL media. Cells not treated with *S. purpurea*, were treated with 25 microL 63% ethanol, 5% glycerol solution (ethanol/glycerol carrier)/mL media. Cells were labeled with [^35^S]-methionine/cysteine Protein Label Mix (Perkin-Elmer) at 4 hpi, as previously described [25]. Cell lysates were analyzed on 12% polyacrylamide gels by SDS-PAGE, dried down on Whatmann filter paper and analyzed by autoradiography.

### Real-time PCR

HeLa cells were mock infected or infected with VACV at an MOI of 10 for 15 min, washed 2× with media, and treated with 25 microL *S. purpurea* extract/mL media. At 4 hpi, total RNA was isolated by the Qiagen RNeasy Mini kit according to the manufacture's protocol. Early RNA levels were quantitatively determined by real-time PCR using specific primers for VACV-E3L mRNA. RNA concentrations were all equalized and real-time PCR was performed with 1 or 10 microL of total RNA.

### Viral core *in vitro* transcription

VACV virion cores were isolated as previously described [26]. Cores were incubated with the indicated amounts of *S. purpurea* or ethanol/glycerol carrier. *In vitro* transcription assays were performed as previously described in the presence of [^35^S]-UTP [26]. A no template reaction was performed by excluding the virion cores. All reactions were done in the presence of Superase-In RNase inhibitor (Ambion). Completed transcription reactions were spotted onto glass-fiber filters, washed 3-times in 10% TCA, and radioactivity was measured by scintillation counting.

## Results

### *Sarracenia* prevented VACV replication

**Figure 1:**
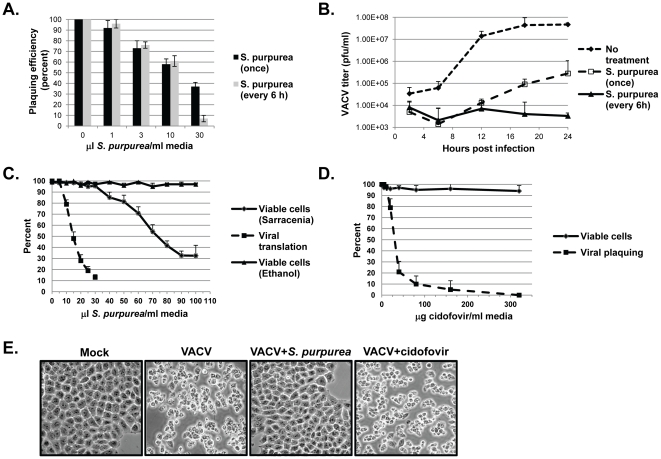
The effect of *S. purpurea* extracts on VACV replication. A) RK-13 cells were infected with 150 pfu of VACV followed by the addition of the indicated concentration of *S. purpurea* extract to the cell culture media. Cells were treated one-time only with the extract (black bars) or every 6 hours with fresh extract (gray bars). After 48 hours, plaques were visualized and quantified. Error bars represent standard deviation (n = 3). B) RK-13 cells were infected with VACV at an multiplicity of infection (MOI) = 10 followed by addition of ethanol/glycerol carrier (closed diamonds) or the addition of 25 microL *S.purpurea* extract/ml media (open squares and closed triangles). Cells were treated one-time only with the extract (open squares) or every 6 hours with fresh extract (closed diamonds). Cells were harvested at the indicated times and viral titers determined. Error bars represent deviation between assays (n = 2). C) For viral translation levels (closed squares), HeLa cells were infected with VACV at an MOI = 10 followed by the addition of the indicated concentrations of *S. purpurea* extract/ml media. At 6 HPI, cell lysates were prepared, the VACV E3L protein detected by Western blot, and quantified. For cell viability, HeLa cells were treated with the indicated concentrations of *S. purpurea* extract (closed diamonds) or ethanol/glycerol carrier (closed triangles) for 6 hours and the number of viable cells determined by a trypan blue exclusion assay. D) For viral plaque formation (closed squares), HeLa cells were infected with VACV (approx. 200 pfu) followed by the addition of the indicated concentrations of cidofovir to the media. After 48 hours, plaques were visualized and quantified. Error bars represent standard deviation (n = 3). For cell viability, HeLa cells were treated with the indicated concentrations of cidofovir (closed diamonds) for 48 hours and the number of viable cells determined by a trypan blue exclusion assay. E) HeLa cells were mock-infected or infected with VACV at an MOI = 10 followed by the immediate addition of media, 25 microL *S. purpurea* extract/ml media, or 320 microg cidofovir/ml media. At 4 HPI, the cell monolayers were photographed.

Initial analysis was done to measure the ability of *S. purpurea* extract to inhibit poxvirus infections *in vitro.* We examined the ability of *S. purpurea* to prevent vaccinia virus (VACV) plaque formation in cells treated immediately after infection with increasing amounts of extract, administered once or every six hours, for a total of 24 hours. We observed a dose dependent reduction in VACV plaquing efficiency associated with increasing amounts of *S. purpurea* extract (Fig. 1a). To ensure *S. purpurea* was responsible for the decrease in plaquing efficiency, cells were treated with 63%∶32%∶5% ethanol∶distilled water∶glycerol (carrier for the *S. purpurea* extract) prior to infection. This carrier treatment did not affect VACV plaquing efficiency (data not shown).

Next we assayed for the ability of VACV to replicate in cells under single-cycle conditions, treated once or every six hours, with *S. purpurea* extract. Treatment with *S. purpurea* began immediately following VACV infection and viral titers were determined every six hours. *S. purpurea* treatment resulted in a dramatic decrease in VACV replication, when compared to the untreated cells (Fig. 1b). A single treatment of *S. purpurea*, caused a 100–1000 fold reduction in VACV replication throughout the course of the infection, however some viral replication was still observed. In cells treated with fresh extract every six hours, a 10,000-fold decrease in VACV replication was observed. Multiple treatments with *S. purpurea* completely abolished VACV replication since titers did not increase over the course of the infection. In cells treated with the carrier, VACV replicated to levels similar to that seen in untreated cells (data not shown). To further determine the efficacy of using *S. purpurea* to treat a poxvirus infection, we determined the selectivity index (SI) associated with the extract. In *S. purpurea* treated cells, the dose required to inhibit VACV replication 50% (EC_50_) was 10–15 microL/mL, while the dose which induced 50% cytotoxicity (CC_50_) was 70–75 microL/mL, resulting in a SI of approximately 5–7 (Fig. 1c). Previous studies have demonstrated that cidofovir (CDV), an already proven treatment for poxvirus infections, has an SI of 6–6.2 in HFF cells [15]–[18]. To compare our results to those of cidofovir, VACV-infected HeLa cells were treated with cidofovir and viral inhibition and cellular toxicity measured (Fig. 1d). Our results demonstrated an EC_50_ for cidofovir of approximately 30 microg/ml, however in cidofovir-treated HeLa cells, no significant toxicity was observed at concentrations up to 640 microg/ml (shown to a dose of 320 microg/ml). This lack of cellular toxicity was similar to that reported previously for cidofovir treated BSC-40 cells [33].

To further compare viral inhibitory activity of the *S. purpurea* extract to cidofovir, the inhibition of viral-induced cytopathic effect (CPE) was evaluated. As shown in Fig. 1e, VACV infection led to significant CPE by 4 HPI (compare Mock to VACV). In a similar infection treated with *S. purpurea*, viral CPE was completed inhibited (Fig. 1e). When the infection was treated with cidofovir, CPE was still observed (Fig. 1e).

### *Sarracenia* inhibited replication early

**Figure 2:**
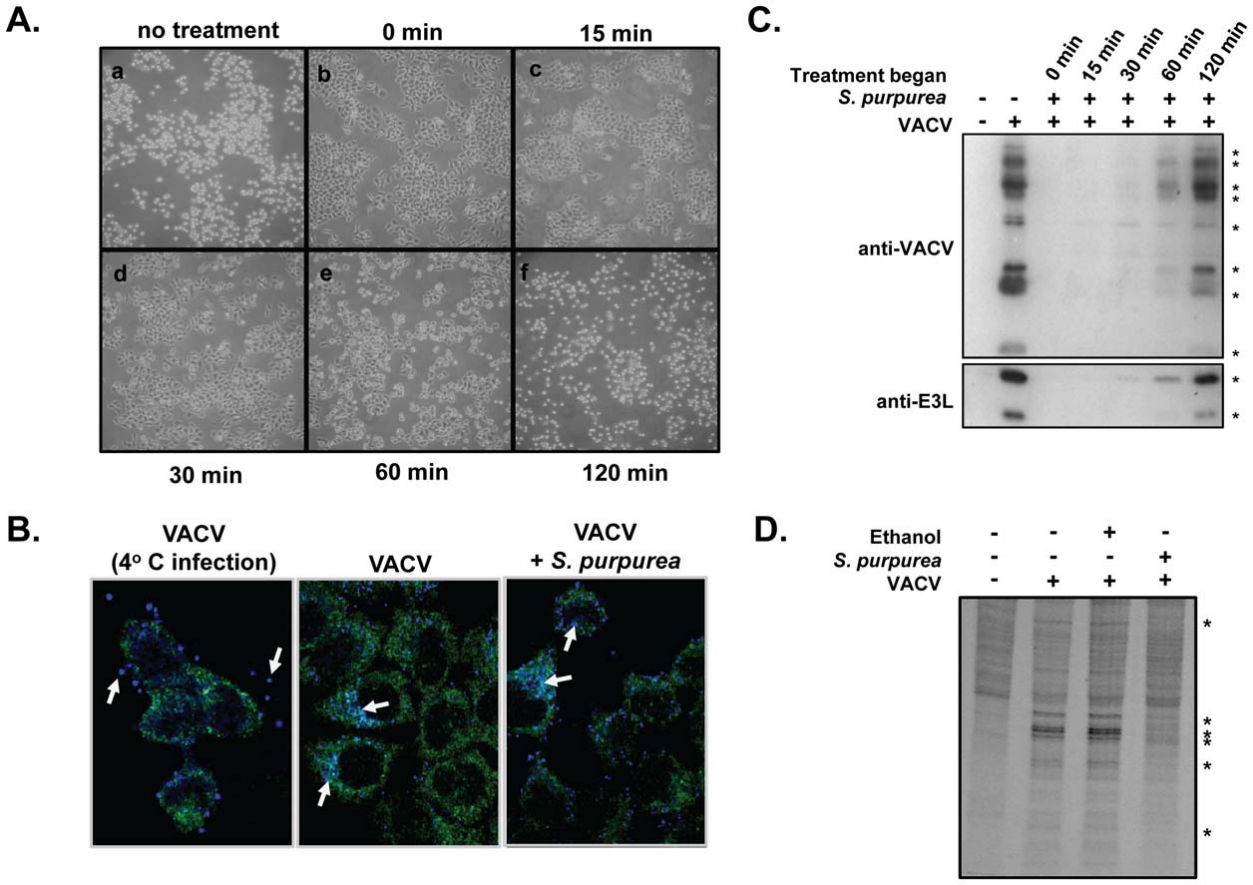
The effect of *S. purpurea* extracts on VACV induced CPE and protein synthesis. A and C) HeLa cells were infected with VACV at an MOI = 10 followed by the addition of 25 microL *S. purpurea* extract/ml media immediately (0 min) or at 15, 30, 60 or 120 min post infection. ‘No treatment’ cells received ethanol/glycerol carrier only. For (A), at 6 HPI, the cell monolayers were photographed. For (C), at 3 HPI, cell lysates were prepared and the VACV E3L protein or total VACV proteins detected by Western blot. * indicate the position of VACV proteins and the VACV-E3L protein. Duplicate experiments were done at 6 HPI (not shown) B) Hela cells were infected with a VACV construct expressing cyan fluorescent protein fused to the viral A5 core protein. In the first panel, the infection was maintained at 4°C. For the middle and last panel, the infection was done at 37°C, in the absence or presence of *S. purpurea*, respectively. D) HeLa cells were mock infected or infected with VACV at an MOI = 10 followed by the addition of 25 microL *S. purpurea* extract/ml media or ethanol/glycerol carrier. At 4 HPI, the cell monolayers were radiolabelled with [^35^S]-methionine, cell lysates prepared, proteins separated by SDS-PAGE, and visualized by autoradiography. * indicate the position of VACV proteins.

The results in Fig. 1e suggest that cidofovir and *S. purpurea* are acting on different targets in the VACV replication cycle and that *S. purpurea* may be inhibiting virus replication early in the replication cycle prior to the induction of CPE. In order to understand the mechanism of antiviral activity associated with *S. purpurea*, various treatment schedules were tested. In cells treated with a single dose of *S. purpurea* overnight prior to infection with VACV followed by washing, no inhibition of VACV replication was observed, suggesting the extract does not induce a cellular antiviral component (data not shown). Moreover, treating a purified VACV stock with *S. purpurea* did not affect replication of the virus, indicating that the extract does not have a direct affect on free virus particles (data not shown). To determine when *S. purpurea* treatment was most effective at preventing VACV replication, we examined the ability of *S. purpurea* to prevent VACV induced CPE when added at various times post-infection. In this assay, cells were infected with VACV and then treated with *S. purpurea* at the indicated times post-infection. At 6 hpi, the cells were examined for VACV induced CPE. In untreated cells, significant VACV induced CPE, specifically cell rounding, was observed (Fig. 2a). However, cells treated at 0, 15, and 30 minutes post-infection (mpi) with *S. purpurea* showed no or low levels of CPE. In cells treated at 60 and 120 mpi, substantial CPE was observed These results are in agreement with Fig. 1e that *S. purpurea* was likely targeting an early component in VACV replication (ie. viral uptake or early viral transcription/translation).

Using a VACV construct in which the core A5 protein was fused to cyan fluorescent protein, we were able to monitor viral uptake into the cell. As shown in Fig. 2b, when the VACV infection was done at 4°C, virus particles remained localized to the periphery of the cell. When the infection was done at 37°C, the majority of virus particles were observed within the cytoplasm. When a similar infection at 37°C was done in the presence of *S. purpurea*, a similar viral localization to the cytoplasm was observed (Fig. 2b). This suggests that *S. purpurea* treatment was not inhibiting VACV uptake into the cell.

We examined if early viral protein synthesis was inhibited following *S. purpurea* treatment. VACV-infected cells were treated with *S. purpurea* at various times post infection and cell lysates were assayed by Western blot using antibodies directed against total VACV proteins or the early VACV protein, E3L. VACV protein synthesis at 3 and 6 hpi in the cells treated at 0, 15, and 30 minutes post infection with *S. purpurea*, was greatly reduced (Fig. 2c). Notably, *S. purpurea* reduced early VACV protein synthesis, as evidenced by the absence of the VACV-E3L protein. In contrast, *S. purpurea* treatment had only marginal affects on viral protein levels when added at 60 and 120 minutes post infection. Cells were also [^35^S]-methionine metabolically labelled to compare viral protein synthesis to cellular protein synthesis following *S. purpurea* treatment. As predicted based on the cellular toxicity data (Fig. 1c), *S. purpurea* inhibited viral protein synthesis, whereas cellular protein synthesis remained unaffected (Fig. 2d). Collectively, these data indicate *S. purpurea* treatment was effective at preventing VACV early protein accumulation and acted at a point between viral uptake and early viral protein synthesis.

### *Sarracenia* inhibited VACV transcription

**Figure 3:**
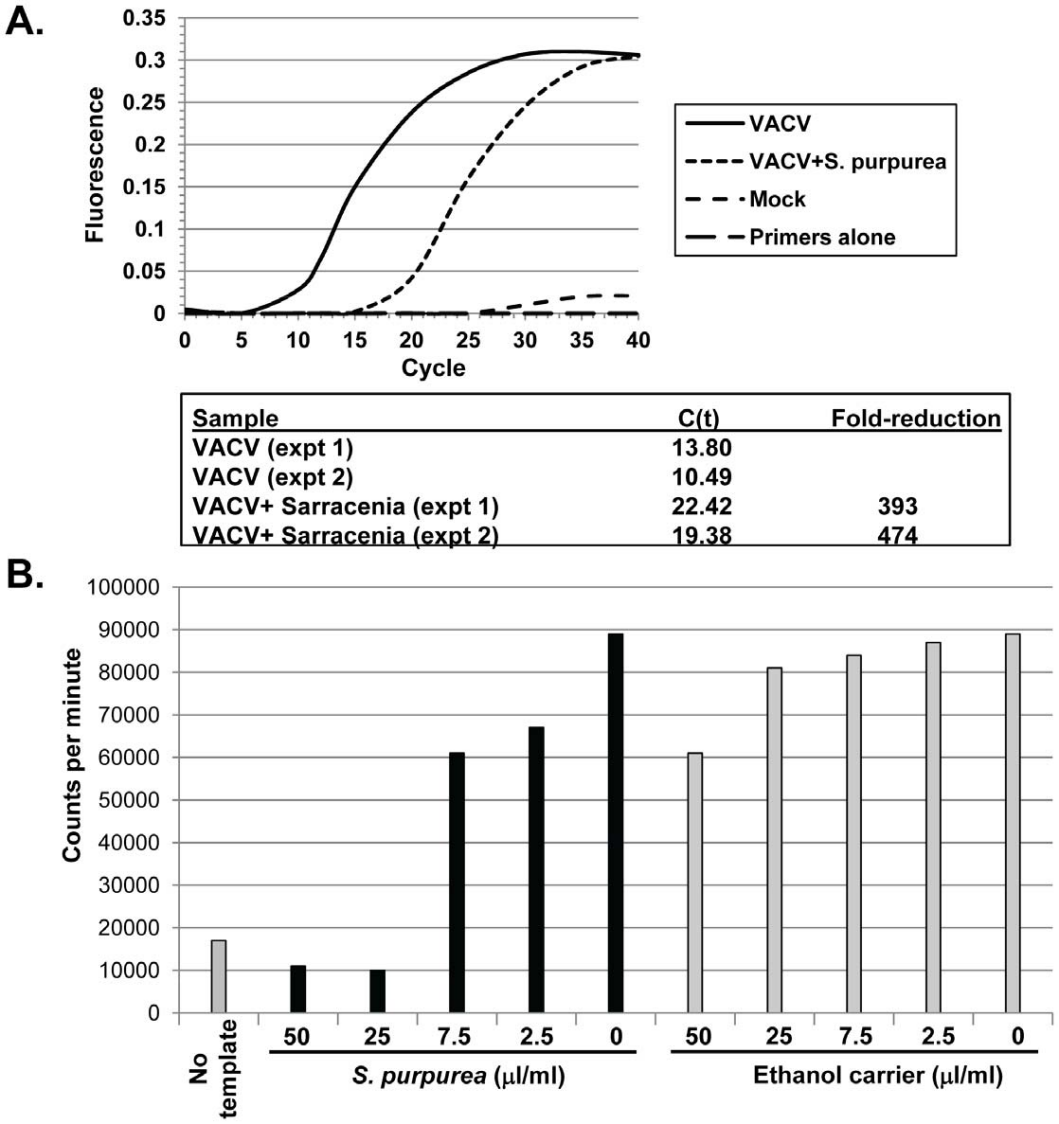
The effect of *S. purpurea* extracts on VACV transcription *in vivo* and *in vitro.* A) HeLa cells were infected with VACV at an MOI = 10 followed by the addition of 25 microL *S. purpurea* extract/ml media. At 4 HPI, total RNA was isolated and VACV-E3L RNA levels determined by real-time PCR. Reactions contained total RNA from mock-infected cells, ethanol/glycerol carrier-treated VACV-infected cells, or *S. purpurea*-treated VACV-infected cells. C(t) values were calculated using manufacturer's software. Graph illustrates data from a representative experiment. C(t) values and fold change from two separate experiments are shown. B) Purified VACV virion cores were incubated in the presence of the indicated concentrations of *S. purpurea* or ethanol/glycerol carrier and [^35^S]-UTP. Newly synthesized RNA products were spotted onto glass-fiber filters, washed in TCA, and quantified by scintillation counting (counts per minute). A no template reaction was performed by excluding the addition of the virion cores.

To further understand the early viral stage affected following *S. purpurea* treatment, we quantified early VACV-E3L mRNA levels in VACV-infected cells using real-time PCR. Total RNA was isolated from cells that were mock-infected, VACV-infected or VACV-infected followed by treatment with a single dose of *S. purpurea* extract. *S. purpurea* treatment resulted in a dramatic reduction of the levels VACV-E3L mRNA present within the infected cells (Fig. 3a). Based on cycle threshold values (C(t)), the levels of early VACV mRNA were decreased by 393–474-fold within the treated cells.

To determine if VACV replication was being blocked at the level of early viral transcription, we performed an *in vitro* transcription assay using purified VACV virion cores and [^3^S]-UTP. The amount of VACV transcription which occurred following treatment with increasing amounts of *S. purpurea* or carrier was measured by quantifying the amount [^3^S]-UTP incorporated into the newly synthesized viral mRNA. As shown, VACV transcription decreased as the amount of *S. purpurea* increased, while the levels of transcription remained relatively equal in the carrier treated cores (Fig. 3b). The amount of *S. purpurea* required to completely inhibit transcription was similar to the dose that prevented VACV replication (Fig. 1b). Addition of *S. purpurea* to VACV cores which had already synthesized RNA did not reduce RNA levels suggesting that the extract did not have intrinsic RNase activity (data not shown).

### *Sarracenia* antiviral activity

**Figure 4:**
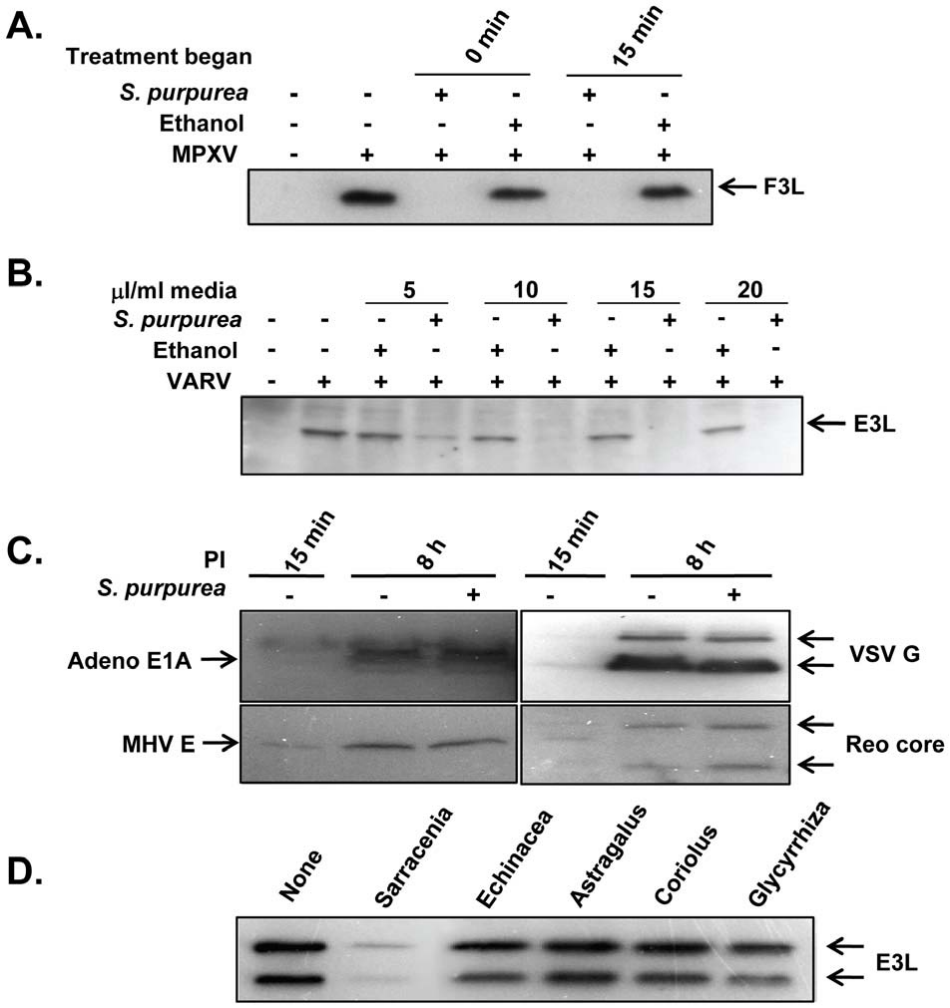
The specificity of *S. purpurea* extracts on *Orthopoxvirus.* A) HeLa cells were mock-infected or infected with monkeypox virus (MPXV) at an MOI = 10 followed by the addition of ethanol/glycerol carrier or 25 microL *S. purpurea* extract/ml media, either 0 or 15 min after infection. At 4 HPI, cell lysates were prepared and the MPXV F3L protein detected by Western blot. B) HeLa cells were mock-infected or infected with variola virus (VARV) at an MOI = 10 followed by the addition of the indicated concentrations of ethanol/glycerol carrier or *S. purpurea* extract to the media at 0 min after infection. At 4 HPI, cell lysates were prepared and the VARV E3L protein detected by Western blot. C) HeLa cells were infected with adenovirus (Adeno), vesicular stomatitis virus (VSV), mouse hepatitis virus (MHV) or reovirus (reo) followed by the addition of ethanol/glycerol carrier or 25 microL *S. purpurea* extract/ml media. At 15 min and 8 hours post-infection, cell lysates were prepared and representative viral proteins detected by Western blot (E1A, G, E, and core proteins, respectively). D) HeLa cells were infected with VACV at an MOI = 10 followed by the addition of ethanol/glycerol carrier (none) or *S. purpurea* extract, *Echinacea* extract, *Astragalus* extract, *Coriolus* extract, or *Glycyrrhiza* extract (25 microL/ml media). At 4 HPI, cell lysates were prepared and the VACV E3L protein detected by Western blot.

To further understand the antipoxvirus activity associated with *S. purpurea*, we decided to analyze the capability of *S. purpurea* to prevent the replication of more virulent members of the *Orthopoxvirus* genus, namely monkeypox virus (MPXV) and variola virus (VARV). Cells were mock-infected or infected with MPXV and subsequently left untreated or treated with *S. purpurea* or carrier at the times indicated. Western blots for the presence of the MPXV-F3L protein (ortholog of the VACV-E3L protein) were performed to determine if a successful MPXV infection had occurred. When treated with the extract at 0 or 15 minutes post infection, *S. purpurea* treatment prevented the accumulation of the MPXV-F3L protein, whereas high levels of MPXV-F3L were detected in the both the untreated and carrier treated cells (Fig. 4a). A similar assay was performed with VARV where cells were mock-infected or infected with VARV and treated with *S. purpurea* or carrier at the dosages indicated (Fig. 4b). Western blots for the presence of the VARV E3L protein indicated a concentration dependent inhibition in the accumulation of the VARV-E3L protein in *S. purpurea* treated infections. In addition, we also determined that *S. purpurea* treatment effectively inhibited rabbitpox virus early protein accumulation (data not shown).

To determine if *S. purpurea* treatment was effective against other viruses outside the Poxviridae family, we analyzed the ability of *S. purpurea* to block the replication of four unrelated viruses, including adenovirus (Adeno) (Adenoviridae, DNA genome), vesicular stomatitis virus (VSV) (Rhabdoviridae, (-) sense RNA genome), mouse hepatitis virus (MHV) (Coronaviridae, (+) sense RNA genome) and reovirus (Reo) (Reoviridae, dsRNA genome). No significant decrease in viral protein synthesis for any of the four viruses tested was detected following treatment with *S. purpurea* (Fig. 4c). We also determined that *S. purpurea* did not affect encephalomyocarditis or VSV plaque formation (data not shown).

Lastly, we wanted to determine if other botanical extracts could prevent VACV replication. In this assay, we chose to examine the effect *Echinacea*, *Astragalus*, *Coriolus*, and *Glycyrrhiza* botanical extracts on VACV protein synthesis and plaque formation. These botanical extracts are thought to be efficacious in the treatment of other microbial infections [28]. Infected cells were treated with each extract and assayed for levels of VACV-E3L protein synthesis. All the botanical extracts contained comparable amounts of non-volatile components as that of the *S. purpurea* extract. As shown, the levels of VACV-E3L decreased dramatically in the *S. purpurea* treated cells (Fig. 4d). However, the cells treated with the four other botanical extracts contained levels of VACV-E3L similar to that seen in the untreated cells. Furthermore, VACV plaquing formation was unaffected by the other botanical extracts (data not shown).

## Discussion

Here we report on the *in vitro* characterization of antipoxvirus activity associated with an *S. purpurea* extract which was historically reported to prevent symptoms associated with a smallpox infection [3], [4], [6], [7], [10], [12]. During the 1800's, patients were treated with *S. purpurea* after the symptoms for smallpox had already appeared and as a preventive during epidemics. Historical reports stated that “the effects were so…speedy and beneficial as to leave no doubt that they were due to the *Sarracenia*” [8]. “The disease continued very severe until (*S. purpurea*) was administered, and became entirely changed in its severity after the administration…, the effect being due to (*S. purpurea*) alone” [1]. In this study, we demonstrated that *S. purpurea* extracts were able to effectively inhibit viral replication and the viral-induced cytopathic effects of various *Orthopoxviruses*. The data supports that *S. purpurea* effectively inhibited MPXV and VARV replication similarly to VACV, and points to the relevance of using VACV as a model of the more virulent MPXV and VARV. At doses where virus replication was inhibited, little to no cellular toxicity was observed.

In regards to broad-spectrum antiviral activity, the data supports that the antiviral activity associated with *S. purpurea* was at least partially specific to poxviruses in comparison to other non-related viruses tested. Since *S. purpurea* treated cells were still able to support the replication of other virus families, it further supports that *S. purpurea* treatment does not disrupt the intracellular environment of the cell. For VACV, a single treatment was fairly effective at preventing viral replication, but partial replication soon recovered, likely due to a breakdown or utilization of the active component(s) within the extract. However, treating the cells with fresh *S. purpurea* every six hours completely abolished the replication of VACV. This correlates well with how patients were treated in the past where the treatment regime involved taking 4–6 doses of the extract per day [11]. Our data supports that extracts of *S. purpurea* effectively inhibit the replication of VACV, MPXV and VARV *in vitro*. This activity toward *Orthopoxviruses* is consistent with the historical reports of *S. purpurea* as a therapy against smallpox infections. The goal of this study was to characterize anti-poxvirus activity of *S. purpurea* extracts in order to verify and evaluate the botanical material under similar preparation methods as that done historically in the 1800's. Collectively, the data suggests that *S. purpurea* targets early viral transcription leading to an inhibition in viral replication. In comparison to other botanicals, the data demonstrate that the anti-poxvirus activity of the *S. purpurea* extract was not necessarily shared by other botanicals. Based on the results presented, future work will involve fractionation and identification of the active anti-poxvirus constituent(s) present in the *S. purpurea* extracts. Cidofovir (CDV) is a broad spectrum antiviral with activity against several DNA viruses, including poxviruses. Currently, CDV is the only licensed parenteral drug with antipoxvirus activity, but its clinical use is governed by its Investigational New Drug (IND) status [30]. Since CDV leads to nephrotoxicity and must be administered intravenously, CDV has not been sought as the standardized therapy to treat poxvirus infections [20]. CMX001, synthesized by coupling CDV to hexadecyl propanedial alkoxykanol, is currently being investigated as an alternative to CDV due to increased bioavailability after oral administration and predicted lack of nephrotoxicity [31]. Another compound, ST-246 (Tecovirimat), has been shown to be highly efficacious in the treatment of poxviruses. ST-246 can be delivered orally and has been reported to be nontoxic within an animal model [21], [32]. To date, ST-246 and CMX001 are the most promising antipoxvirus compounds. A report issued by the Institute of Medicine, as well as the 2009 WHO Advisory Committee on Variola Virus Research, suggested that due to the increased susceptibility of the human population to smallpox, *at least* two anti-variola virus drugs with different mechanisms of action should be developed in order to provide greater protection in the event of a smallpox epidemic [22]. ST-246 and CMX001 have potent efficiencies and target different points in the viral replication cycle, but single point mutations in the poxvirus genome can confer resistance to these drugs. Therefore, additional anti-poxvirus therapies would certainly aid in protecting the human population and increase the repertoire of treatments available.

As a measurement of the efficacy associated with *S. purpurea*, the SI was determined to be similar to that previously reported for CDV [15], [16]. Although anecdotal, when extracts of *S. purpurea* were used in the late 1800's, no severe side effects were ever reported following treatment [1], [2], [7], [8], [9], [12]. *Sarracenia* extracts have a modern use in the southern United States as a laxative for dyspepsia. In patients ingesting *S. purpurea*, diuretic responses were often observed with significantly elevated levels of limpid urine, but no other physiological effects reported [5]. Further controlled studies will be necessary to fully understand the safety profile of *S. purpurea*. It is possible that *S. purpurea* may not be as cytotoxic in a human as compared to a cell-culture system. Indeed, extracts from the related species, *S. flava*, have been shown to possess antitumor activity [29]. It is possible, since the cells used in this study are established cell lines, the cell toxicity observed may be due to this antitumorigenic response. Indeed, further testing with animal models and purification of the active component(s) will be necessary to provide the information required to determine the overall safety and efficacy of using *S. purpurea* to treat *Orthopoxvirus* infections. Extensive animal studies to further characterize the safety profile and antipoxvirus activity associated with *S. purpurea* will be conducted, but insurmountable challenges exist in conducting human trials associated with VACV, MPXV and VARV infections. Although human trials to measure overall safety associated with administration of *S. purpurea* can be done, antipoxvirus studies cannot be conducted. Similar challenges have also been observed with studies associated with the recently identified antipoxvirus drug, ST-246, however this drug has been used for the treatment of VACV-vaccination related complications in humans [32].

**Figure 5:**
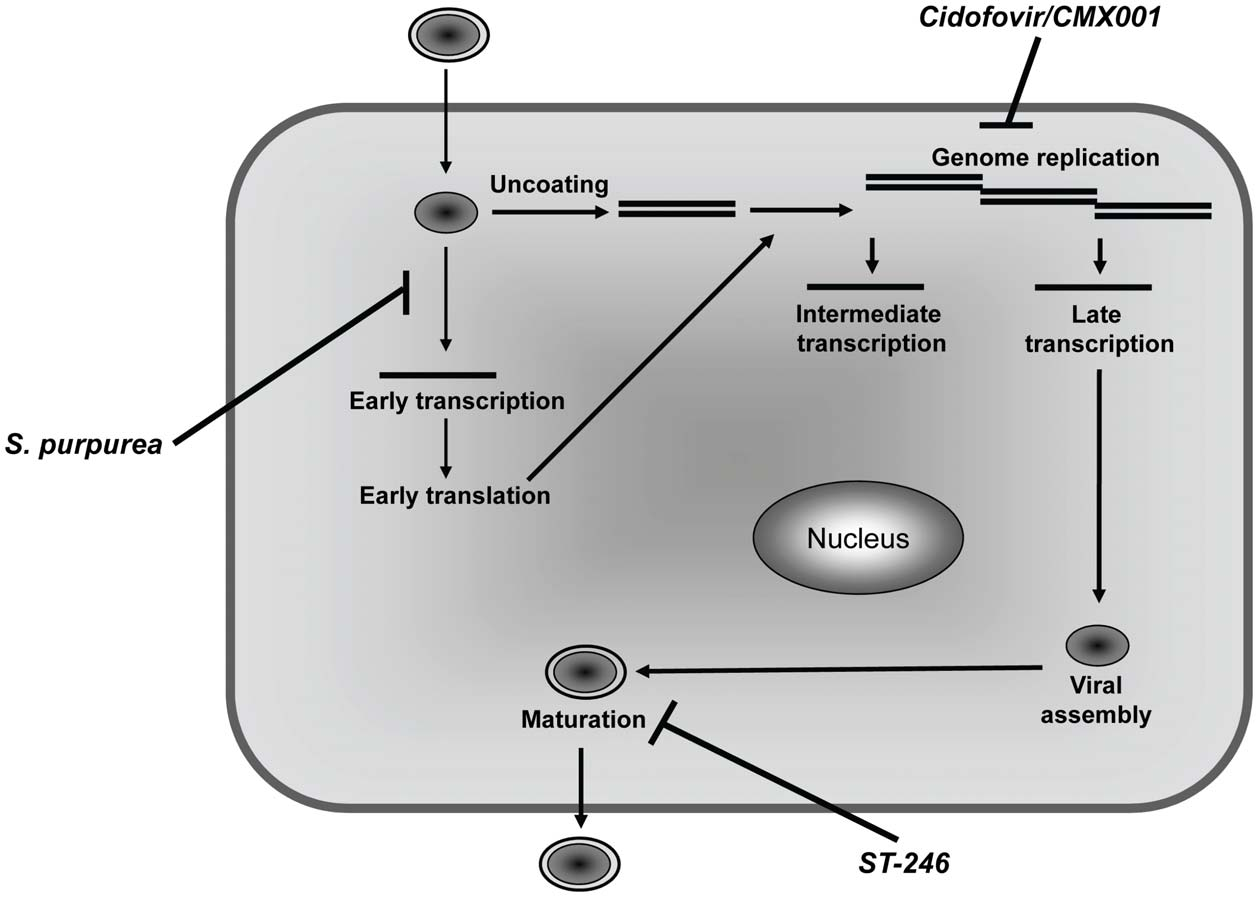
Mechanism of action of poxvirus therapeutics. Illustration indicates the general replication cycle of VACV. The previously shown targets of known antipoxvirus compounds, cidofovir and ST-246, are shown, as well as the presumptive target of the *S. purpurea* extract.

It has been suggested that having multiple antipoxvirus therapeutics which work by distinctly different mechanisms would be beneficial during a smallpox epidemic or emergence of novel poxvirus pathogens [22]. Many of the initially developed anti-poxvirus compounds, like CDV, were nucleoside analogues [30]. Alternately,ST-246 acts by preventing extracellular virus formation which is essential for dissemination of the virus (Fig. 5) [21]. In this study, we were able to demonstrate that *S. purpurea* specifically affected early viral transcription, the first antiviral agent to be characterized that targets poxviruses at this point in the replication cycle (Fig. 5). Although solely based on *in vitro* studies, our work supports the potential of *S. purpurea* as an additional treatment for poxvirus infections, either individually or in combination with other known antivirals.
